# Olive Leaf Extracts from Three Italian Olive Cultivars Exposed to Drought Stress Differentially Protect Cells against Oxidative Stress

**DOI:** 10.3390/antiox13010077

**Published:** 2024-01-07

**Authors:** Luca Cerri, Sara Parri, Maria Celeste Dias, Angela Fabiano, Marco Romi, Giampiero Cai, Claudio Cantini, Ylenia Zambito

**Affiliations:** 1 Department of Pharmacy, University of Pisa, Via Bonanno 33, 56126 Pisa, Italy; l.cerri3@student.unisi.it (L.C.); angela.fabiano@unipi.it (A.F.); ylenia.zambito@unipi.it (Y.Z.); 2 Department of Life Sciences, University of Siena, Via Mattioli 4, 53100 Siena, Italy; sara.parri@student.unisi.it (S.P.); romi5@unisi.it (M.R.); 3 Centre for Functional Ecology, Department of Life Sciences, University of Coimbra, Calçada Martim de Freitas, 3000-456 Coimbra, Portugal; celeste.dias@uc.pt; 4 Institute for BioEconomy (IBE), National Research Council (CNR), Strada Provinciale Aurelia Vecchia 49, 58022 Follonica, Italy; claudio.cantini@ibe.cnr.it; 5 Research Centre for Nutraceutical and Healthy Foods “NUTRAFOOD”, University of Pisa, Via del Borghetto 80, 56124 Pisa, Italy

**Keywords:** OLE, HUVECs, polyphenols, antioxidants, oleuropein, drought stress

## Abstract

Olive leaves are an abundant by-product of olive oil production. Olive leaf extracts (OLEs) are rich in polyphenols, which can be used for health benefits. As polyphenols are the main antioxidant molecules in plants, plants typically increase their polyphenol content when exposed to drought stress. However, the phenolic profile of OLEs can vary in relation to the origin and variety of the plant material. In this work, olive leaf extracts from three different Italian olive cultivars (Giarraffa, Leccino, and Maurino) both exposed and not exposed to drought stress were studied in terms of antioxidant properties and profile, intestinal permeation, and protection against oxidative stress of human umbilical vein endothelial cells (HUVECs), since HUVECs are considered a model to study a wide range of diseases. OLEs from stressed Maurino and Giarraffa plants showed the highest increase in antioxidant capacity compared to controls. The phenolic profile of Maurino’ was mainly increased by water deficit, with a large increase in the compounds oleuropein and luteolin-7-*O*-rutinoside. All tested extracts exposed to a water deficit protected HUVECs against oxidative stress by reducing ROS production, and this effect was more pronounced in OLEs from Giarraffa and Maurino exposed to drought stress compared to all other extracts. Finally, OLE from the stressed Giarraffa group showed a higher apparent permeability of antioxidant molecules than that of Maurino.

## 1. Introduction

The olive tree (*Olea europaea* L.) is one of the most widespread plants; in fact, more than 8 million hectares of olive trees are cultivated worldwide, 98% of them in the Mediterranean area [1]. The plant is mainly cultivated for the economically and culturally rewarding production of olive oil. Even though olive leaves have high nutraceutical properties, both branches and shoots from pruning and harvesting are discarded in large quantities without considering the possibility of them being a valuable source of nutraceuticals. For every liter of olive oil produced, about 6 kg of leaves are discarded [2] and are usually burned or ground and spread on the fields [3].

Numerous reports have described the importance of olive leaf by-products as a rich source of bioactive compounds [4]. The antioxidant activity of olive leaf extracts (OLEs) is due to the presence of polyphenols [5], which makes them an ideal candidate in the medical, cosmetic, and pharmaceutical fields [6]. For example, OLEs’ protective effects against oxidative stress were found in endothelial cells [7], in renal cells exposed to cadmium [8], and in bronchial epithelial cells affected by cystic fibrosis [9]. Oleuropein is the most abundant compound in OLE [10] and is thought to be primarily responsible for its pharmacological effects. However, OLE contains a wide variety of flavonoids, which is the most abundant group of polyphenols in olive leaves. Therefore, the use of whole extracts may provide greater antioxidant capacity and health benefits than isolated compounds, thanks to the synergistic effects of all the polyphenols present [11].

In plants, polyphenols are involved in tolerance to various abiotic stresses, such as heat, drought, flood, light, salinity, and heavy metals [12]. Thanks to its long-term adaptation to the dry conditions of the Mediterranean, the olive tree is considered a woody plant model to study drought stress responses [13]. Several studies report the variation of phenolic compounds in response to drought [14,15,16]. The increase in the content of total polyphenols as a consequence of drought stress contributes to an increase in the antioxidant properties of the extracts [17]. However, the phenolic profile of OLE varies depending on the origin and the variety of the plant material [18]. The Italian National Register has catalogued as many as 700 olive cultivars (as defined in D.M. 7521 4 March 2016). 

We previously analyzed three Italian olive cultivars under drought stress conditions, selected on the basis of their geographical origin [19]. The cultivars Leccino’ and ‘Maurino’ are thought to be derived from a local oleaster of central Italy [20]. The former is cultivated worldwide thanks to its resistance to the bacterium *Xylella fastidiosa* [21]; the latter is mainly distributed in central Italy. Giarraffa is genetically distant from other Italian olive cultivars and was probably introduced from Spain and Morocco [20]. It is mainly cultivated in Sicily. The cultivars showed different physiological responses to drought.

In this work, OLEs from Giarraffa, Leccino, and Maurino, both exposed and not exposed to drought stress, were studied in terms of antioxidant properties and profile, protection against the oxidative stress of HUVEC cells, and intestinal permeation. HUVECs are an excellent model to investigate a wide range of diseases, such as cardiovascular and metabolic diseases; therefore, several studies have evaluated antioxidant molecules from natural products on HUVECs through in vitro experiments related to vascular dysfunction [22]. In this manuscript, we perform a thorough analysis of the antioxidant capacity of OLEs from three Italian olive cultivars to determine the one with the most relevant effects on HUVEC cells. In order to better discriminate between the cultivars, the latter were drought stressed and the phenolic profile of OLEs was determined to unravel their different protection against reactive oxygen species (ROS) in human cells. Since, in this study, drought stress was primarily used to discriminate the potential antioxidant response of olive cultivars, we focused on molecules that provide protection against ROS in HUVEC cells rather than on their biochemical role in olive tree metabolism. The outcome of this research could potentially convert the agricultural by-product of olive leaves into a high-value nutraceutical compound.

## 2. Materials and Methods

### 2.1. Materials

Olive leaf extracts of the Giarraffa (OLE-G), Leccino (OLE-L), and Maurino (OLE-M) varieties and the extracts obtained from the same cultivars subjected to water deficit (OLE-GS, OLE-LS, OLE-MS) were collected from the Life Sciences Department of the University of Siena, Siena (SI), Italy. The HUVEC cells were purchased from Lonza (Basel, Switzerland). MCDB 131 Medium was purchased from Gibco-Thermo Fischer (Waltham, MA, USA). Fetal bovine serum (FBS), L-glutamine, and heparin sodium salt from porcine intestinal mucosa were purchased from Sigma-Aldrich (Darmstadt, Germany). Human FGF-basic and Human EGF (animal free) were purchased from Peprotech (Waltham, MA, USA). Cell proliferation reagent (WST-1) was provided by Roche diagnostic (Mannheim, Germany). The fluorescent probe 2,7-dichloro-dihydro-fluorescein diacetate, acetyl ester (CM-H2DCFDA), was provided by Invitrogen (Carlsbad, CA, USA). Gallic acid (GA), ferrous chloride, and Folin–Ciocalteu reagent were purchased from Merck (Darmstadt, Germany).

#### Leaf Sampling and Stress Condition

Leaves were collected from certified 18-month-old olive trees (*Olea europaea* L., cultivars Leccino, Maurino and Giarraffa) provided by Spoolivi (Società Pesciatina di Orticoltura, Pescia, PT, Italy). Growing conditions, drought treatments, and the plants’ water status have been described in detail by Parri et al. (2023) [19]. Briefly, for each cultivar, 10 plants were completely water deprived for 4 weeks (OLE-GS, OLE-LS, and OLE-MS for the Giarraffa, Leccino, and Maurino water deficit stress groups, respectively) and 10 plants were fully watered (OLE-G, OLE-L, and OLE-M for the Giarraffa, Leccino, and Maurino control groups, respectively). For each experimental group, a pool of about 40 leaves was sampled at t0, t1, t2, t3, and t4, corresponding to the start of withholding watering and the first, second, third, and fourth weeks of water deprivation, respectively. Leaves were collected, immediately frozen at −80 °C, and used for the following analysis.

### 2.2. Determination of the Antioxidant Capacity and Polyphenol Content

The extracts were prepared following the procedure described by De la Ossa (2021) [23] but slightly modified. In detail, frozen leaves (1 g) were ground in liquid nitrogen and the powder was resuspended in 3 mL of 70% acetone. Samples were homogenized with Ultra-Turrax T-25 basic (IKA^®^-Werke GmbH & Co. KG, Staufen Germany) for 3 min and sonicated with an ultrasonic bath (Transsonic T 460/H Elma, Singen, Germany) for 20 min. The homogenate was centrifuged at 4000× *g* for 5 min at 4 °C. The supernatants, which contained the antioxidant extracts, were collected and used for the antioxidant power and polyphenol content determination. Another aliquot of the same supernatants was filtered through a 0.45 µm cellulose acetate membrane filter (Sartorius, Göttingen, Germany), frozen, and freeze-dried for 48 h (freeze dryer LIO 5P, 5 Pascal, Italy). The lyophilized olive supernatants were used for the cell viability test via WST-1 assay, ROS production analysis, and assessment of the permeation of antioxidants contained in the OLE across excised rat intestine.

### 2.3. Ferric Ion-Reducing Antioxidant Power (FRAP)

The FRAP method was carried out to determine the antioxidant capacity [24]. For each reaction, 20 μL of extract were mixed with 2040 μL of 300 mM acetate buffer pH 3.6, 200 μL of 10 mM TPTZ (2,4,6-tripyridyl-s-triazine), and 200 μL of 20 mM FeCl_3_. After 1 h of incubation at 37 °C, the absorbance of the samples was measured using a UV-Vis spectrophotometer (wavelength set at 563 nm). The absorbance values were interpolated on a standard curve using known ferrous sulfate solutions. The antioxidant power of each group was expressed in mmol of ferrous chloride equivalent per 100 g of matter.

### 2.4. Folin–Ciocalteu Method

The total polyphenol content was determined by the Folin–Ciocalteu colorimetric assay [25]. For each reaction, 500 μL of extract were mixed with 3950 μL of distilled water, 250 μL of Folin–Ciocalteu reagent, and 750 μL of a sodium carbonate-saturated solution (Na_2_CO_3_) for each reaction. After a 30 min incubation at 37 °C, the absorbance of each sample was measured at 795 nm using a UV-Vis spectrophotometer. The absorbance value was interpolated using a standard curve of a known gallic acid solution. Total phenolic content was measured in milligrams of gallic acid equivalent (GAE) per 100 g of matter.

### 2.5. Leaf Metabolite Extraction and Ultra-High-Performance Liquid Chromatography–Mass Spectrometry

Only the leaves sampled at t4 were used for this extraction. Approximately 4 g of frozen olive leaves were dried for 7 days at 40 °C. The leaves were then grinded in a small mill and the powder was used for metabolite extraction. Two extraction cycles were performed with methanol (1:10, w:v). The dry extract obtained (50 mg) was dissolved in 1 mL of pure and filtered methanol (nylon membrane with a pore size of 0.2 µm, Whatman, Merck, Darmstadt, Germany). A sample with a concentration of 10 mg/mL was prepared and 4 µL were injected into a Thermo Scientific Ultimate 3000RSLC Dionex (Waltham, MA, USA) equipped with a Dionex UltiMate 3000 RS diode array detector coupled with a mass spectrometer operating in negative ion mode. A Hypersil GOLD column (1.9 µm particle diameter, Thermo Scientific, Lenexa, KS, USA) was used. Analysis and compound identification were performed as described in Dias et al. (2019) [26]. The UV-Vis spectral data were collected between 250 and 500 nm, and the chromatogram profile was recorded at 280 nm. A semi-quantitative analysis was performed via peak integration through the standard external method. The identification of the peaks was performed by comparing the retention times, UV-Vis spectra, and spectral data obtained from the reference compounds. The detection and quantification limits were determined through calibration curves calculated with the reference compounds (quercetin for flavonoids and oleuropein for secoiridoids). The calibration curves were obtained via injection of different concentrations of quercetin (y = 4 × 10^6^x − 390882 and R^2^ = 0.99, where x is the amount of the compound expressed in mg/mL and y is the peak area obtained in the chromatogram) and oleuropein (y = 10^6^x − 6948 and R^2^ = 0.98, where x is the amount of the compound expressed in mg/mL and y is the peak area obtained in the chromatogram) using the same conditions as for the sample analysis. The results are expressed in mg of the compound/g of tissue DW and presented as the mean ± standard deviation of 3 independent analyses per sampling time and treatment.

### 2.6. Cell Viability Test by WST-1 Assay

The viability of the HUVECs was assessed with the WST-1 assay. For this purpose, 2 × 10^4^ cells per well were seeded in 96-well plates and placed in an incubator for 24 h at 37 °C and 5% CO_2_. Fifteen hours after seeding, the cells were incubated for 4 h with the OLEs at concentrations in the 1–50 µg/mL range. The samples to be tested were dissolved in fresh medium and filtered through 0.45 µm cellulose acetate filter prior to contact with the cells. Following the 4 h treatment, the cells were washed with fresh medium to completely remove any residual extract, then incubated at 37 °C for 2 h with WST-1 reagent diluted 1:10. The amount of formazan produced was evaluated at 450 nm. After washing, some of the cells were subjected to oxidative stress by incubating them for 1 h with 500 µM H_2_O_2_. In this case, the WST-1 reagent was added after completely removing H_2_O_2_ from the wells through suitable washing.

### 2.7. ROS Production

ROS production was evaluated as previously described [17]. During the last 30 min of treatment with OLEs or H_2_O_2_, the HUVECs were incubated with the fluorescent probe 2,7-dichloro-di-hydro-fluorescein diacetate, acetyl ester (CM-H2DCFDA), dissolved in PBS at a concentration of 10 µM in the dark at room temperature. At the end of the experiment, ROS production was detected by measuring the increase in fluorescence at an excitation of 488 nm and an emission of 510 nm.

### 2.8. Permeation of Antioxidants Contained in OLE across Excised Rat Intestine

A well-known procedure [27,28] authorized by the scientific–ethical committee of the Italian university and the Italian Ministry for Universities and Research was carried out. Briefly, under veterinary supervision, the intestinal mucosa was excised from non-fasted male Wistar rats with a weight of between 250 and 300 g. Longitudinal strips were obtained from the intestine by cutting, rinsed to remove luminal contents, and then mounted in Ussing-type cells with an exposed surface area of 0.78 cm^2^ while preserving the underlying muscle layer. After 20 min of equilibration at 37 °C, OLE-G, OLE-GS, OLE-M, or OLE-MS dispersed in phosphate buffer of pH 7.4 (0.13 M) was added to the apical chamber. The experiment was performed with OLEs at the same extract concentration of 3 mg/mL and the same GAE content of 0.16 mg/mL. Three mL of fresh phosphate buffer of pH 7.4 (0.13 M) were added to the acceptor chamber. To ensure oxygenation of tissue and stirring, both compartments were bubbled with a mix of Clioxicarb (95% O_2_ plus 5% CO_2_ mix). The transport of OLEs from apical to basolateral was studied. At 30 min intervals for a total of 150 min, 1 mL of sample was withdrawn from the acceptor and replaced with fresh pre-thermostated medium. The amount of antioxidant molecules that permeated was determined by analyzing the samples via the Folin–Ciocalteau method.

### 2.9. Statistical Analysis

All data are expressed as mean ± standard deviation (SD). When not stated otherwise, six independent replicates were performed for each experiment. Data were tested for normality of distribution using the Shapiro–Wilk test. Significant differences between the extracts analyzed were determined by one-way ANOVA. When ANOVA showed *p* ≤ 0.05, a post-hoc test (Bonferroni correction) was performed. *p* ≤ 0.05 was considered to indicate a significant difference.

## 3. Results

### 3.1. Antioxidant Capacity and Polyphenol Content

**Figure 1:**
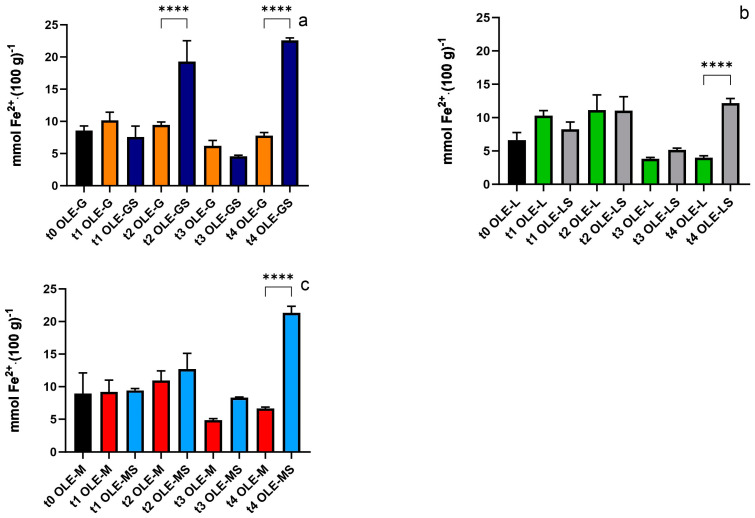
Antioxidant capacity of (**a**) OLE-G and OLE-GS, (**b**) OLE-L and OLE-LS, and (**c**) OLE-M and OLE-MS from t0 to t4. Asterisks indicate a statistically significant difference (****: *p* < 0.001) between the stressed group and its respective control. In each panel, unstressed and stressed samples are indicated by different colors, regardless of the time of analysis.

Figure 1 shows the antioxidant capacity of leaves collected at different time points. After two weeks of stress, the first difference between OLE-G and OLE-GS appeared (Figure 1a). However, as the drought stress progressed, the antioxidant capacity increased in all cultivars: OLE-GS, OLE-LS, and OLE-MS were significantly different from their respective controls at t4. At this time point, OLE-GS and OLE-MS showed a higher antioxidant value (22.6 mmol Fe^2+^ (100 g)^−1^ and 21.3 mmol Fe^2+^ (100 g)^−1^, respectively) compared to OLE-LS (12.2 mmol Fe^2+^ (100 g)^−1^).

**Figure 2:**
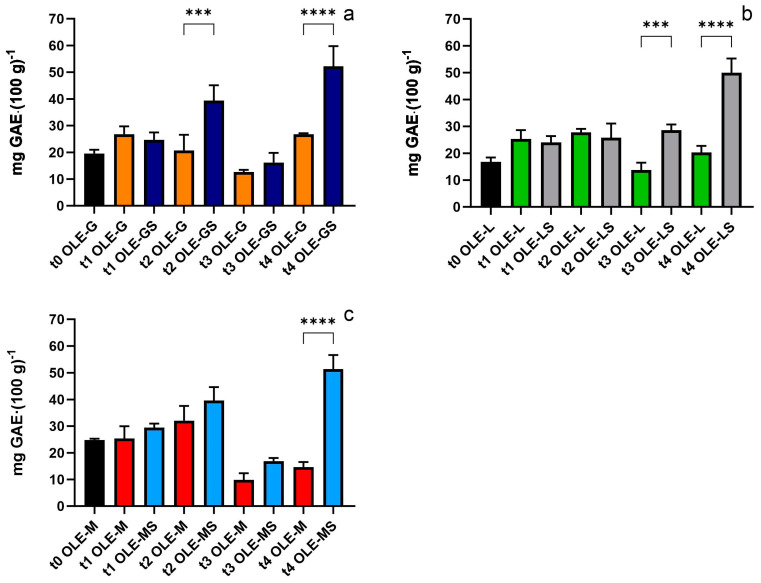
Polyphenol content of (**a**) OLE-G and OLE-GS, (**b**) OLE-L and OLE-LS, and (**c**) OLE-M and OLE-MS from t0 to t4. Asterisks indicate a statistically significant difference (****: *p* < 0.001; *** *p* < 0.001) between the stressed group and its respective control.

The polyphenol content of each group during the experimental period is shown in Figure 2. The polyphenol content increased after four weeks of drought stress, when all the stressed groups differed significantly from their respective control. As shown in Figure 2a, the polyphenol content in OLE-GS had already increased by t2 (39.4 mg GAE (100 g)^−1^), but the highest value was reached at t4 (52.2 mg GAE (100 g)^−1^). Despite the different antioxidant capacities, the polyphenol content in OLE-GS, OLE-LS, and OLE-MS was very similar at t4 (51.3, 49.9, 50.3 mg GAE (100 g)^−1^, respectively).

The strongest response, in terms of both antioxidant capacity and polyphenol content, was observed after four weeks of stress. Therefore, for all cultivars, leaves collected at t4 were selected for UHPLC characterization and analysis on human cells.

### 3.2. OLE Phenolic Characterization

In total, 16 compounds were quantified: 3 secoiridoids and 13 flavonoids. There were some differences between the extracts of the three olive varieties. In OLE-L, only one secoiridoid (aldehyde form of decarboxyl elenolic acid) was detected, whereas the flavonoids chrysoeriol-7-*O*-glucoside and luteolin-7-*O*-glucoside were not detected in either OLE-L or OLE-LS. In the same retention time (12.1 min), the extracts from Giarraffa contained luteolin-7-*O*-rutinoside instead of 7-*O*-glucoside, as occurred in the extracts from Leccino and Maurino. The total amount of phenolic compounds was higher in OLE-LS (46.1 mg/g DW) and OLE-MS (79.1 mg/g DW) compared to their respective controls (40.7 mg/g DW in OLE-L and 39.9 mg/g in OLE-M). In OLE-LS, the increase was mostly due to the higher amount of detected secoiridoids (10.0 mg/g DW) compared to OLE-L (4.3 mg/g DW). OLE-MS showed a higher detected amount of both secoiridoids (27.7 mg/g DW) and flavonoids (51.4 mg/g DW) compared to OLE-M (3.2 mg/g DW and 36.7 mg/g DW, respectively). Conversely, OLE-GS showed a decrease in the total amount of phenolic compounds detected (29.4 mg/g DW) compared to OLE-G (33.2 mg/g DW) due to a lower amount of flavonoids having been detected (30.4 mg/g DW in OLE-G, 26.7 mg/g DW in OLE-GS).

The extracts from OLE-MS presented the largest (*p* < 0.05) amounts of oleuropein and oleuropein aglicone, dihydroquercetin, luteolin-7-*O*-rutinoside, and chysoeriol-7-*O*-glucoside, and OLE-M presented the largest (*p* < 0.05) amount of diosmetin (Table 1). In turn, OLE-L showed the largest (*p* < 0.05) amount of the aldehyde form of decarboxyl elenolic acid and OLE-LS the largest (*p* < 0.05) amount of apigenin-*O*-dideoxyhexoside-hexoside, apigenin-7-*O*-rutinoside, and luteolin-7-*O*-glucoside (Table 1).

**Figure 3:**
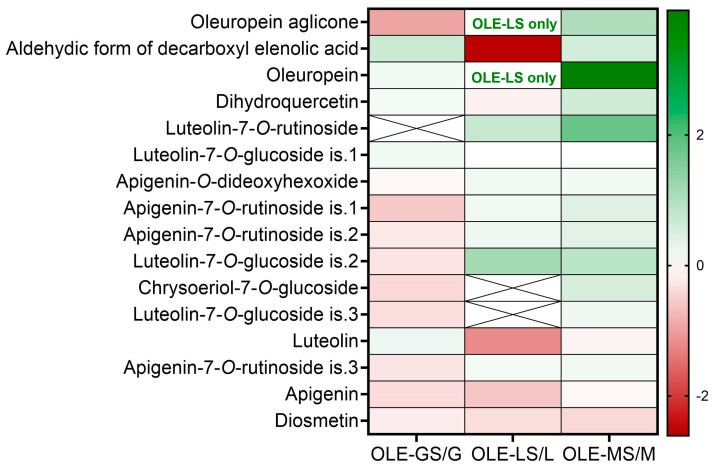
Heat map of the fold changes (log_2_ (stressed/control)) in phenolic metabolites of Giarraffa (OLE-GS/OLE-G), Leccino (OLE-LS/OLE-L), and Maurino (OLE-MS/OLE-M).

Figure 3 shows the heat map of the fold changes of the phenolic metabolites extracted from the three cultivars. In the Giarraffa cultivar, drought stress caused, in general, a decrease in the phenolic content of OLE-GS/OLE-G, except for the secoiridoid aldehyde form of decarboxyl elenolic acid and oleuropein and the flavonoids dihydroquercetin, luteolin-7-*O*-glucoside is. 1, and luteolin, which slightly increased. In contrast, a more heterogeneous response profile was observed in OLE-LS/OLE-L, with a similar number of compounds having increased and decreased due to drought stress. The phenolic pool of the Maurino cultivar mostly increased with water deficit, with a large increase in oleuropein and luteolin-7-*O*-rutinoside.

### 3.3. Cell Viability Test

**Figure 4:**
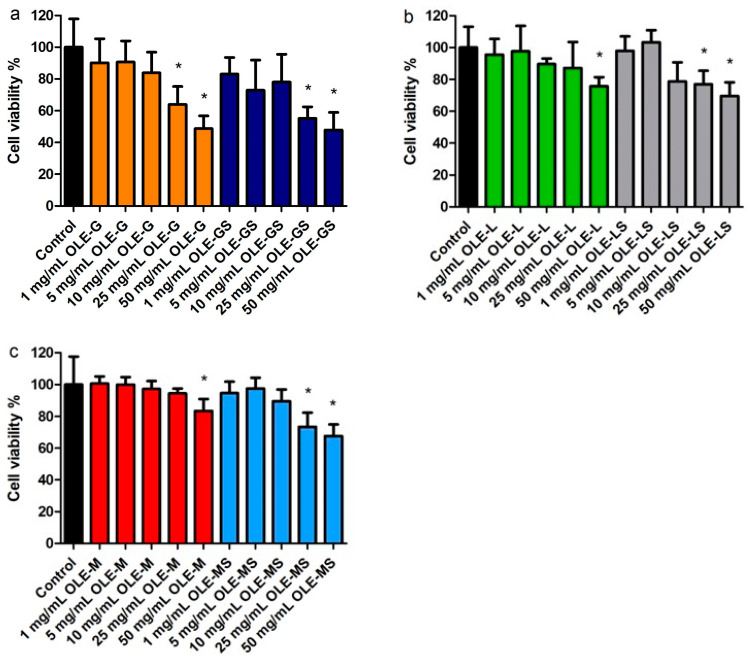
HUVEC viability after 4 h of incubation with (**a**) OLE-G and OLE-GS, (**b**) OLE-L and OLE-LS, and (**c**) OLE-M and OLE-MS in culture medium. Data are expressed as % viable cells in reference to 100% control (untreated cells). Data are reported as mean ± SD (*n* = 6). *, Significantly different from control (*p* < 0.05).

Figure 4 shows the viability of HUVECs treated with increasing concentrations of OLE-G and OLE-GS (Figure 4a), OLE-L and OLE-LS (Figure 4b), and OLE-M and OLE-MS (Figure 4c). The data in Figure 4a show that OLE-G and OLE-GS were cytotoxic at concentrations above 10 µg/mL. Figure 4b,c show that the olive leaf extracts of the Leccino and Maurino varieties had a low cytotoxicity at all of the concentrations tested. Therefore, the concentration of 10 µg/mL was chosen to carry out the subsequent experiments.

### 3.4. OLE Protective Effect from Oxidative Stress

**Figure 5:**
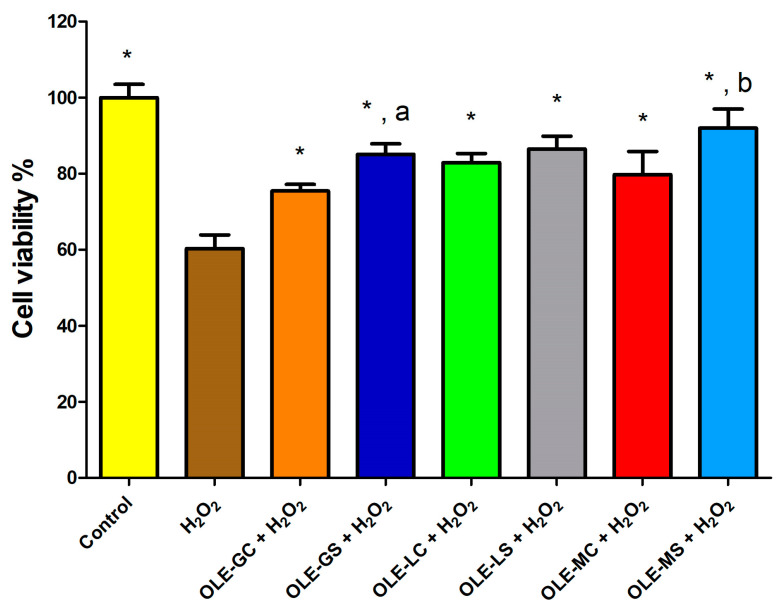
HUVEC viability after 2 h pre-treatment with OLE-G, OLE-GS, OLE-L, OLE-LS, OLE-M, and OLE-MS (10 μg/mL) in culture medium and subsequent 1 h treatment with 500 μM H_2_O_2_. Data are expressed as % viable cells compared to negative control (H_2_O_2_). *, Significantly different from H_2_O_2_ (*p* < 0.05); a, significantly different from OLE-G (*p* < 0.05); b, significantly different from OLE-M (*p* < 0.05).

With this test, the influence of the treatment with OLEs on the viability of HUVECs after oxidative stress induced by H_2_O_2_ was evaluated. The OLEs were obtained from plants both subjected to and not subjected to water deficit stress. Figure 5 shows data on the protection of HUVECs from oxidative damage after 2 h of pre-treatment with OLEs at a non-toxic concentration of 10 µg/mL and subsequent treatment with 500 µM of H_2_O_2_ for 1 h. The data show that oxidative stress significantly reduced the number of viable cells compared to control (cells with medium only). Pre-treatment of HUVECs with all the extracts under study at a concentration of 10 µg/mL reduced H_2_O_2_ cytotoxicity significantly. Apparently, the extracts from the Giarraffa and Maurino varieties that were subjected to water deficit stress (OLE-GS and OLE-MS) were significantly more effective than the corresponding extracts from olive trees that were not subjected to water deficit stress (OLE-G and OLE-M).

**Figure 6:**
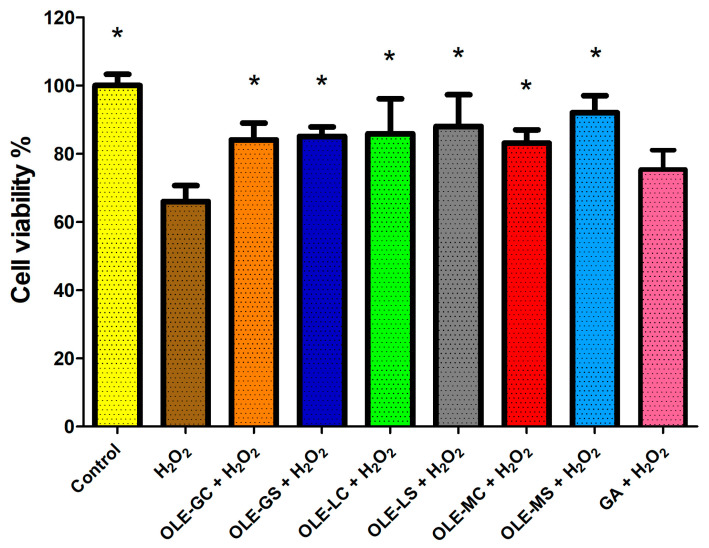
HUVEC viability after 2 h pre-treatment with OLE-G, OLE-GS, OLE-L, OLE-LS, OLE-M, OLE-MS, and GA (0.5 μg/mL GAE) in culture medium and subsequent 1 h treatment with 500 μM H_2_O_2_. Data are expressed as % viable cells compared to negative control (H_2_O_2_). *, Significantly different from H_2_O_2_ (*p* < 0.05).

Figure 6 shows data on the protection of HUVECs from oxidative damage after 2 h of pre-treatment with OLEs and gallic acid (GA) at the same polyphenol concentration of 0.5 µg/mL and subsequent treatment with 500 µM of H_2_O_2_ for 1 h. Also in this case, oxidative stress significantly reduced the number of viable cells compared to the control (cells with medium). HUVEC cell pre-treatment with all extracts at a concentration of 0.5 µg/mL of GAE showed a significant protective effect against H_2_O_2_ oxidative damage compared to GA pre-treatment. However, there were no statistical differences between the various OLEs tested.

### 3.5. OLE Antioxidant Activity as Assessed by ROS Production

**Figure 7:**
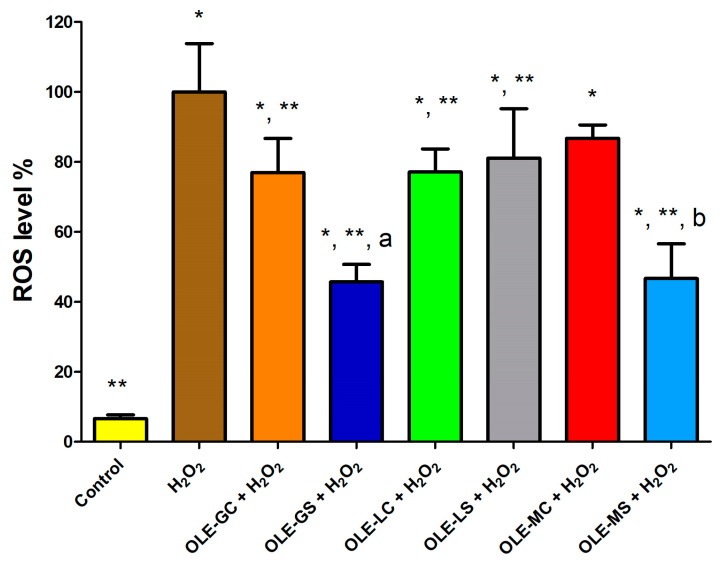
Reactive oxygen species (ROS) level in HUVECs treated with OLE-G, OLE-GS, OLE-L, OLE-LS, OLE-M, and OLE-MS (10 μg/mL) in culture medium and subsequent treatment with 500 μM H_2_O_2_ for 1 h. Data are expressed as % ROS production on the basis of cells simply treated with H_2_O_2_. *, Significantly different from control (*p* < 0.05); **, significantly different from H_2_O_2_ (*p* < 0.05); a, significantly different from OLE-G (*p* < 0.05); b, significantly different from OLE-M (*p* < 0.05).

Figure 7 shows the data for ROS production in HUVECs pre-treated and not pre-treated with the OLEs of interest and then subjected to oxidative stress. As can be seen, ROS production after cell treatment with H_2_O_2_ was significantly higher than ROS production in cells incubated with control (plain medium). All extracts tested, except OLE-M, significantly reduced ROS production compared to cells treated with H_2_O_2_. It was apparent that OLE-GS and OLE-MS significantly reduced ROS production compared to OLE-G and OLE-M, respectively. On the other hand, no significant differences were observed between OLE-L and OLE-LS. The results obtained with this test are in perfect agreement with those reported in Figure 4. These results can be attributed to the fact that OLE-GS and OLE-MS had a significantly higher content of polyphenols and antioxidants than all the other extracts and were not significantly different from each other.

**Figure 8:**
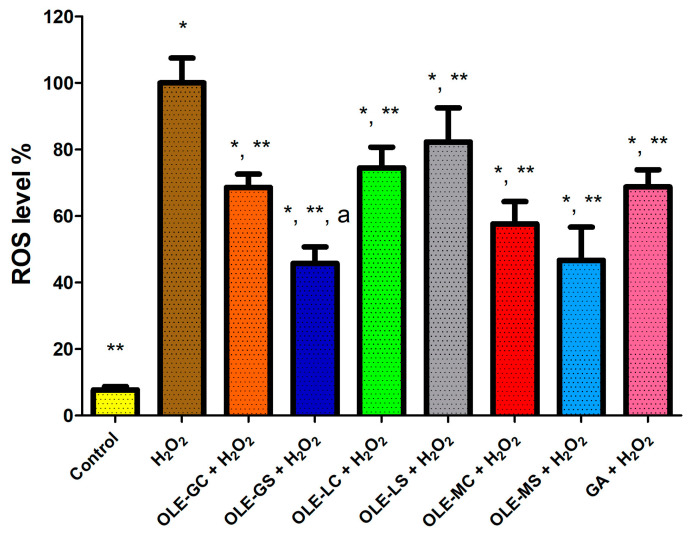
Reactive oxygen species (ROS) production in HUVECs treated with OLE-G, OLE-GS, OLE-L, OLE-LS, OLE-M, OLE-MS, and GA (0.5 μg/mL GAE) in culture medium and subsequent treatment with 500 μM H_2_O_2_ for 1 h. Data are expressed as % ROS production compared to 100% (cell treated with H_2_O_2_). *, Significantly different from control (*p* < 0.05); **, significantly different from H_2_O_2_ (*p* < 0.05); a, significantly different from OLE-G and GA (*p* < 0.05).

ROS level was also evaluated in HUVECs with OLEs at the same GAE concentration (0.5 μg/mL GAE). GA was also tested as a positive control (Figure 8). All extracts tested significantly reduced the ROS level compared to the H_2_O_2_-treated cells. Surprisingly, ROS in OLE-GS-treated cells was significantly lower than that in OLE-G- and GA-treated cells. This result demonstrates that extracts from olive leaves subjected to water deficit stress contained antioxidant molecules with higher reducing power than those extracted from non-stressed olive trees.

### 3.6. Permeation of Antioxidants Contained in OLEs across Excised Rat Intestine

**Figure 9:**
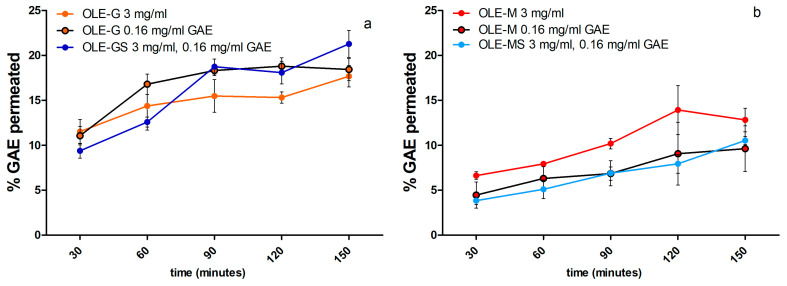
Data on the permeation of antioxidant molecules contained in (**a**) OLE-G and OLE-GS and in (**b**) OLE-M and OLE-MS in phosphate buffer (pH 7.4, 0.13 M) at the same concentration of extract (3 mg/mL) or at the equivalent gallic acid concentration (GAE) of 0.16 mg/mL across the excised jejunal rat epithelium (*n* = 3).

The epithelium of the excised rat jejunal tract was selected among known ex vivo intestinal models for studies of the permeability of antioxidant molecules because its tight junctions are similar in size and number to those of the human jejunum [29]. Only the OLEs extracted from the Maurino and Giarraffa varieties, which showed a greater antioxidant ability than that of the Leccino variety, were tested for permeation through the intestinal epithelium. The OLEs were tested while keeping constant either the concentration of the extract or the amount of polyphenols present in the extracts, expressed in mg of GAE per mL. Figure 9a,b report the percentage of antioxidant molecules permeating through the intestinal epithelium over time. As can be seen, all the OLEs tested had the same permeation profile regardless of the concentration or amount of antioxidant molecules applied. However, by comparing the data in Figure 9a with those in Figure 9b, it can be seen that the OLEs obtained from the Giarraffa variety had a significantly higher permeability than those obtained from the Maurino variety. These results, together with those shown in Figure 8, allow us to conclude that the OLEs from the Giarraffa variety were more permeable and, in particular, those obtained from plants subjected to water deficit stress had higher antioxidant activity.

## 4. Discussion

*Olea europaea* L. is one of the most abundant, ancient, and economically valuable crops in the Mediterranean. Olive leaves are an unavoidable by-product of olive oil production, accounting for 25% of the dry weight of the total pruning residue (more than 6 kg/L of olive oil produced) [30]. Olive leaves are known to be rich in phenolic derivatives, mainly consisting of simple phenols, flavonoids, and secoiridoids, which may have various beneficial biological effects thanks to their antimicrobial, antioxidant, antiviral, and cardioprotective properties [11]. The accumulation of phenolic compounds is a well-known response to various abiotic stresses. As drought will be one of the major challenges in the Mediterranean region, research on cultivar-specific antioxidant properties under controlled or stressful conditions may be useful to identify which cultivars have the highest antioxidant content and potential health benefits, thus turning olive leaves from agricultural waste into a health or pharmaceutical product.

In this work, the antioxidant effects of olive leaf extracts of the olive cultivars Giarraffa, Leccino, and Maurino were compared with those of the same leaves subjected to water deficit stress.

As reported in other studies [31,32], drought stress increased the antioxidant responses of olive plants. In the present study, the antioxidant capacity (FRAP) and total phenols (TPC) reached their highest levels in the drought-stressed plants by the end of the experimental period after four weeks of total water deprivation. At this time point, in OLE-GS and OLE-MS, the increase in the antioxidant capacity response was reflected by an accumulation of polyphenols. OLE-LS maintained a lower antioxidant capacity, but the polyphenol content increased. In fact, the antioxidant activity of an extract depends not only on the quantity of the polyphenols but also on the type and the synergistic interactions that occur [33]. The chemical structure heavily determines the antioxidant properties of phenolics: Catechol moieties, multiple hydroxyl groups, and conjugation with electron-donating groups at the 4-position of the aromatic ring are factors that positively influence antioxidant activity [33,34]. UHLC-MS characterization of the phenolic compounds present in the leaf extracts revealed differences between cultivars on the basis of water supplementation. Oleuropein, a 3,4-dihydroxyphenylethanol (hydroxytyrosol) ester with a-glucosylated elenolic acid, is commonly reported as the main component of olive phenolic extracts [7,15,33], and it is well known for its pharmacological effects related to its free radical-scavenging properties [11]. In this study, oleuropein was found to be the main component of leaf phenolic extracts in OLE-MS and OLE-LS. According to the antioxidant capacity calculated for single phenolic compounds by Xie et al. (2015) [33] and Benavente-Garcia et al. (2000) [34], OLE-MS is rich in high-performance compounds such as oleuropein, dihydroquercetin, and flavon-7-glucosides of both luteolin and apigenin, with a probable consequent decrease in the free form of these flavones. Similarly, OLE-LS showed an increase in the content of these compounds compared to OLE-L; however, the constant level of high antioxidant performance of dihydroquercetin and the lower increase in phenolics under stress conditions may have contributed to the lower FRAP response of OLE-LS compared to OLE-MS. Synergistic effects should be taken into account when considering the results of OLE-GS. The lower levels of all phenolic compounds detected in OLE-GS compared to OLE-G (Figure 3) do not justify its higher antioxidant capacity (Figure 1a). As suggested by Dias et al. (2019) [26], this may have been due to several reasons: It is possible that the stress condition increased the use in radical scavenging more than it affected the phenolic synthesis, in which case the antioxidant phenolic molecules did not increase in quantity. Alternatively, the molecules detected could have been in combination with some other antioxidant compounds not detected by LC-MS analysis.

The antioxidant activity of the olive leaf extracts was evaluated on HUVEC cells. All OLEs showed no cytotoxicity at the concentrations analyzed (1–50 μg/mL); however, it can be observed in Figure 4 that the viability decreased slowly with increasing concentration, especially with the OLE from Giarraffa both when subjected and not subjected to water deficit stress. Similar results were found in [23] and indicate that a high concentration of polyphenols present in olive extract exerts a cytotoxic effect, whereas a low concentration increases cell proliferation, as already demonstrated [35,36]. Olive leaves demonstrate scavenging activity against multiple ROS and could display cardiovascular protection ability. The ability of polyphenols presents in OLEs to inhibit ROS production was evaluated in this study. All OLEs tested, in particular, the OLEs from Giarraffa and Maurino subjected to water deficit stress, were able to reduce ROS production compared to untreated cells. Considering that these extracts had a higher antioxidant activity and polyphenol content than the others, these differences could be related to a synergic effect between the present antioxidant compounds [37]. Therefore, the protective effect of the OLEs could be related to a determined concentration and combination of antioxidants present in the extract. Finally, in this study, we evaluated the ability of antioxidants present in the OLEs from Giarraffa and Maurino to cross the excised intestinal wall. As reported in Figure 9b, the OLE from Giarraffa had a significantly higher permeability than the OLE from Maurino. In fact, a permeation of total polyphenols at 20% of the applied dose (0.16 mg/mL GAE) was observed in the OLE from Giarraffa, whereas the OLE from Maurino demonstrated permeation at about 10%. These results indicate that OLE from Giarraffa is more permeable through the intestine compared to OLE from Maurino probably because it depends on the permeation ability of the single molecules it contains. Indeed, various factors should be considered in the study of the transport of bioactive compounds in the intestine, such as concentration and degradation processes [38].

## 5. Conclusions

The extracts subjected to water deficit stress studied in this work, OLE-GS, OLE-MS, and OLE-LS, have been shown to demonstrate antioxidant activity on HUVECs, thus providing protection against oxidative damage. Among all the extracts, the OLEs from Giarraffa and Maurino showed better performance on HUVECs than the OLE from Leccino. Although OLE-GS showed a decrease in the total amount of phenolic compounds compared to the same extract not subject to water deficit stress, it had more antioxidant capacity and greater permeability across the rat intestine than OLE-MS. The results obtained allow us to conclude that the antioxidant activity of extracts obtained from plants subjected to water stress on HUVEC cells is greater than that of extracts obtained from control plants, encouraging us to continue studies on OLEs subjected to water deficit.

## Figures and Tables

**Table 1:** Phenolic profile (mg/g DW) of OLE-G, OLE-GS, OLE-L, OLE-LS, OLE-M, and OLE-MS extracts. Values are mean ± standard deviation (*n* = 3–4). Rt—retention time; nd—not detected; is.—isomer. For each compound, different letters denote significant difference (*p* < 0.05) between the values.

Rt (min.)	Compound	[M-H]–(*m*/*z*)	MS^2^ (*m*/*z*) Fragments	OLE-G	OLE-GS	OLE-L	OLE-LS	OLE-M	OLE-MS
	Secoiridoids								
10.3	Oleuropein aglicone	377	197/153	1.140 ± 0.109 ^b^	0.596 ± 0.025 ^d^	nd	1.244 ± 0.022 ^b^	0.875 ± 0.024 ^c^	1.797 ± 0.005 ^a^
10.8	Aldehydic form of decarboxyl elenolic acid	215	197/153/ 171/185	0.475 ± 0.118 ^b^	0.752 ± 0.076 ^b^	4.314 ± 0.681 ^a^	0.708 ± 0.033 ^b^	0.653 ± 0.184 ^b^	0.973 ± 0.014 ^b^
14.4	Oleuropein	539	377/307/275	1.228 ± 0.276 ^c^	1.377 ± 0.106 ^c^	nd	8.095 ± 0.494 ^b^	1.661 ± 0.845 ^c^	24.897 ± 1.353 ^a^
	Flavonoids								
11.9	Dihydroquercetin	303	285/177/ 125	2.506 ± 0.145 ^c^	2.722 ± 0.032 ^c^	2.655 ± 0.014 ^c^	2.391 ± 0.204 ^c^	3.430 ± 0.048 ^b^	5.279 ± 0.089 ^a^
12.1	Luteolin-7-*O*-rutinoside	593	447/285	nd	nd	2.584 ± 0.015 ^d^	4.261 ± 0.167 ^b^	3.008 ± 0.046 ^c^	10.366 ± 0.125 ^a^
12.1	Luteolin-7-*O*-glucoside is. 1	447	287/285	2.029 ± 0.070	2.305 ± 0.171	nd	nd	nd	nd
12.4	Apigenin-*O*-dideoxyhexoside-hexoxide	449	269	1.826 ± 0.023 ^d^	1.726 ± 0.016 ^d^	2.628 ± 0.015 ^b^	2.992 ± 0.015 ^a^	2.250 ± 0.025 ^c^	2.479 ± 0.174 ^b^
12.8	Apigenin-7-*O*-rutinoside is. 1	577	269	3.471 ± 0.125 ^c^	2.350 ± 0.050 ^e^	4.797 ± 0.090 ^b^	5.364 ± 0.071 ^a^	2.688 ± 0.023 ^d^	3.621 ± 0.047 ^c^
13.0	Apigenin-7-*O*-rutinoside is. 2	577	269	2.485 ± 0.102 ^c^	2.099 ± 0.107 ^d^	2.849 ± 0.036 ^b^	3.296 ± 0.199 ^a^	2.283 ± 0.041 ^c^	2.979 ± 0.037 ^b^
13.3	Luteolin-7-*O*-glucoside is. 2	447	285	3.737 ± 0.163 ^bc^	3.035 ± 0.462 ^bc^	2.762 ± 0.233 ^c^	6.135 ± 0.250 ^a^	4.039 ± 0.139 ^b^	7.161 ± 0.638 ^a^
13.5	Chrysoeriol-7-*O*-glucoside	461	299/446	2.198 ± 0.099 ^c^	1.669 ± 0.007 ^d^	nd	nd	2.840 ± 0.112 ^b^	4.111 ± 0.127 ^a^
13.9	Luteolin-7-*O*-glucoside is. 3	447	285	2.215 ± 0.145 ^b^	1.741 ± 0.063 ^c^	nd	nd	2.506 ± 0.173 ^ab^	2.878 ± 0.187 ^a^
15.7	Luteolin	285	285	2.668 ± 0.018 ^c^	3.098 ± 0.290 ^c^	7.160 ± 0.197 ^a^	3.109 ± 0.014 ^c^	4.905 ± 0.119 ^b^	4.546 ± 0.200 ^b^
16.7	Apigenin-7-*O*-rutinoside	577	269	2.000 ± 0.004 ^c^	1.651 ± 0.078 ^d^	2.086 ± 0.034 ^c^	2.262 ± 0.019 ^b^	2.339 ± 0.073 ^b^	2.619 ± 0.046 ^a^
17.4	Apigenin	269	269/225	2.612 ± 0.041 ^d^	2.010 ± 0.077 ^e^	5.982 ± 0.089 ^a^	3.956 ± 0.042 ^b^	2.884 ± 0.012 ^c^	2.701 ± 0.010 ^d^
17.8	Diosmetin	299	284	2.637 ± 0.028 ^c^	2.283 ± 0.049 ^d^	2.877 ± 0.020 ^b^	2.277 ± 0.005 ^d^	3.537 ± 0.047 ^a^	2.674 ± 0.014 ^c^

## Data Availability

Data available on request due to restrictions.
